# Out of Florida: mtDNA reveals patterns of migration and Pleistocene range expansion of the Green Anole lizard (*Anolis carolinensis*)

**DOI:** 10.1002/ece3.324

**Published:** 2012-08-08

**Authors:** Shane C Campbell-Staton, Rachel M Goodman, Niclas Backström, Scott V Edwards, Jonathan B Losos, Jason J Kolbe

**Affiliations:** 1 Department of Organismic and Evolutionary Biology, Museum of Comparative Zoology, Harvard UniversityCambridge, MA, 02138; 2 Department of Biology, Hampden-Sydney CollegeHampden Sydney, VA, 23943; 3 Department of Biological Sciences, University of Rhode IslandKingston, RI, 02881

**Keywords:** Glaciation, lizards, mitochondrial variation, North America, phylogeography

## Abstract

*Anolis carolinensis* is an emerging model species and the sole member of its genus native to the United States. Considerable morphological and physiological variation has been described in the species, and the recent sequencing of its genome makes it an attractive system for studies of genome variation. To inform future studies of molecular and phenotypic variation within *A. carolinensis*, a rigorous account of intraspecific population structure and relatedness is needed. Here, we present the most extensive phylogeographic study of this species to date. Phylogenetic analyses of mitochondrial DNA sequence data support the previous hypothesis of a western Cuban origin of the species. We found five well-supported, geographically distinct mitochondrial haplotype clades throughout the southeastern United States. Most Florida populations fall into one of three divergent clades, whereas the vast majority of populations outside Florida belong to a single, shallowly diverged clade. Genetic boundaries do not correspond to major rivers, but may reflect effects of Pleistocene glaciation events and the Appalachian Mountains on migration and expansion of the species. Phylogeographic signal should be examined using nuclear loci to complement these findings.

## Introduction

Phylogeographic studies examine the spatial distribution of genetic lineages within species and are critical to understanding organismal variation (Avise 2000). By revealing patterns of intraspecific relatedness, they place observations of natural variation and divergence into an evolutionary context, allowing development of hypotheses about their origin.

Previous phylogeographic studies of *Anolis* lizards have revealed high levels of population divergence within widespread species. On islands of the Greater Antilles, deep mitochondrial divergence has been reported for widespread species from Puerto Rico (Rodriguez-Robles et al. 2007), Hispaniola (Glor et al. 2003), Jamaica (Jackman et al. 2002), and Cuba (Glor et al. 2004, 2005; Kolbe et al. 2004; Knouft et al. 2006). This trend has also been well documented in species across the Lesser Antilles (Malhotra and Thorpe 1994, 2000; Schneider 1996; Ogden and Thorpe 2002; Thorpe 2002; Thorpe and Stenson 2003; Thorpe et al. 2005) and species occupying the Amazon Basin of mainland South America (Glor et al. 2001; D'angiolella et al. 2011). In several cases, the degree of mitochondrial divergence between populations is comparable to that observed between species, suggesting that some widespread *Anolis* species may be complexes of allopatrically or parapatrically distributed species. Moreover, divergence in mitochondrial loci is not always congruent with historical boundaries, geographic distance, or morphological variation, suggesting that mitochondrial clades may delimit reproductively isolated units across a species range (Gibbs et al. 2006). For a review of anole phylogeography, see Losos (2009).

*Anolis carolinensis* is a model species for laboratory studies in neurobiology and physiology as well as reproductive behavior and morphology (Lovern et al. 2004). With the recent publication of the green anole genome (Alfoldi et al. 2011), its utility as a model system will be extended to include studies of genome architecture and evolution (Fujita et al. 2011), links between genotypic and phenotypic variation, and the genomic basis of local adaptation and population differentiation. *Anolis carolinensis* is the sole member of its genus native to the United States, where fossil bones dating to the Wisconsinan glacial period have been identified from caves in Florida, Georgia, and Alabama (reviewed in Holman 1995). The current species' range spans the vast majority of the southeastern United States, extending as far north as Tennessee and North Carolina and as far west as eastern Texas. Phylogeographic evidence from other species in this region reveals a substantial influence of glacial history and geographic topology on patterns of relatedness within species (reviewed in Soltis et al. 2006).

Rivers often serve as important genetic boundaries for reptiles and amphibians in the southeastern United States, including the Apalachicola (e.g., Church et al. 2003; Zamudio and Savage 2003; Liu et al. 2006), Tombigbee (Lawson 1987), and the Mississippi (e.g. Austin et al. 2004; Hoffman and Blouin 2004; Moriarty and Cannatella 2004). The Appalachian Mountains have also been identified as a significant barrier to gene flow in the southeastern herpetofauna (e.g., Zamudio and Savage 2003; Austin et al. 2004; Jones et al. 2006). Evidence from southeastern flora and fauna suggest that Pleistocene refugia in Florida (Scott and Upchurch 1982; Riggs 1984; Hayes and Harrison 1992; Ellsworth et al. 1994), the Gulf Coast and Mississippi Valley (Delcourt and Delcourt 1981; Jackson et al. 2000; Swenson and Howard 2005), and the southern Appalachians (Brant and Orti 2003; Church et al. 2003; Austin et al. 2004) have influenced the genetic structuring of species across this region (reviewed in Soltis et al. 2006).

Previous studies of the extent and distribution of genetic variation within *A. carolinensis* indicate little genetic divergence throughout the species' range, with genetic uniformity between Florida, Louisiana, and Texas populations (Webster et al. 1972), small genetic distances between Texas and Georgia (Buth et al. 1980), and a high degree of similarity between Alabama, Texas, and Florida populations (Wade et al. 1983). However, these studies have not provided a comprehensive picture of population-level relationships due to their limited geographic scope and the discrepancy between sampling localities among these studies. Specifically, both Webster et al. (1972) and Buth et al. (1980) only sampled three populations, and although Wade et al.'s (1983) sampling was more extensive, including Tennessee, Louisiana, Texas, Alabama, and Florida, it left much of the Atlantic coast and western portions of the species' range unsampled.

To better inform studies of phenotypic and genomic variation within this species, a comprehensive account of evolutionary history and population structure is needed. Toward this aim, we report the most geographically extensive phylogeographic analysis of *A. carolinensis* to date using samples from field collections, museum collections, and sequences from the NCBI online genetic database (Genbank) representing 37 sites across the species' range and 19 populations of its Cuban progenitor *Anolis porcatus*. Using mitochondrial DNA (mtDNA) sequences we (1) examine phylogenetic relatedness of populations across the species' range and (2) identify geographic factors and historical events that potentially influenced the evolutionary history of the species.

## Materials and Methods

### Sample collection

**Figure 1:**
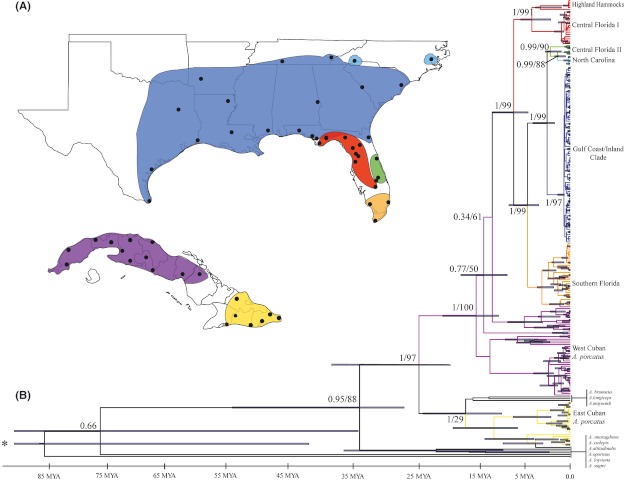
(A) Map of collection sites throughout the *Anolis carolinensis* range in the southeastern United States and *A. porcatus* in Cuba. (B) Consensus Bayesian tree of *A. carolinensis*, *A. porcatus, A. brunneus, A. longiceps, A. maynardi*, and *A. smaragdinus* samples from three independent analyses in BEAST with the application of a molecular clock rate of 1.3% pairwise divergence/million years. *Anolis altitudinalis, A. isolepis, A. loysiana, A. oporinus*, and *A. sagrei* are used as the out-group species. The *x*-axis shows dates of the divergence events in years before present. Clades are labeled to the right and colors correspond to the map. Support values above nodes are posterior probabilities and bootstrap values (PP/BS). Node error bars represent the 95% confidence intervals on each divergence time calculated using a relaxed molecular clock model, and are not calculated for nodes with posterior probability support less than 0.50. The single asterisk (*) marks a truncated error bar with an upper divergence time of 170.5MYA.

During May–June of 2006 and 2007, we collected 29–42 male and female *A. carolinensis* from each of 17 populations throughout the southeastern United States. Collection sites included both natural and human-modified habitats, but did not contain artificial water sources. Due to low population densities, collections were restricted to 10 and 17 lizards from Brownsville, TX and Naples, FL, respectively. We also sampled 3–8 individuals from 10 populations throughout Florida, and obtained 24 samples from museum collections, one individual from North Carolina, and 23 individuals from nine populations in Florida (see Fig. 1A for population sampling and Table S1 for locality information). Previously published ND2 mtDNA sequences for *A. porcatus* and *A. carolinensis* (Glor et al. 2004; Kolbe et al. 2007) as well as sequences of four other closely related species (*Anolis brunneus, A. longiceps, A. maynardi*, and *A. smaragdinus*) were obtained from GenBank.

### Molecular methods

We extracted genomic DNA from liver, tail, or toe tissue using a salt extraction protocol (Sambrook and Russell 2001). We amplified and sequenced a ∼1200 bp fragment of mtDNA including the genes ND2, tRNA^Trp^, and tRNA^Ala^. This entire region was amplified using the primers L4437 (Macey et al. 1997) and H5934 (Glor et al. 2004). Our PCR protocol was as follows: denaturation at 95**°**C for 180 sec, 30 cycles of 95**°**C for 35 sec, 53**°**C for 35 sec, and 70**°**C for 150 sec, and a final extension at 70**°**C for 300 sec. Reaction volume of 30 *u*L included 2 *u*L genomic DNA and a mixture of 47% ddH_2_O, 10% 10× buffer, 10% BSA, 10% 25 mM MgCl_2_, 3% dNTPs, 10% 2 pmol of each primer, and 0.5% *Taq* DNA polymerase. PCR products were purified using ExoSAP-IT (USB Corp., Cleveland, OH). The purified PCR products were used as template for Big Dye® Terminator v.3.1 sequencing reactions (MCLAB, South San Francisco, CA), which were cleaned with Sephadex® (Sigma–Aldrich, St. Louis, MO) and visualized on an ABI 3730 (Applied Biosystems, Foster City, CA).

### Phylogenetic analysis

Sequences were aligned manually using MacClade version 4.0.5 (Maddison and Maddison 2000). We used *Anolis altitudinalis, A. isolepis, A. loysiana, A. oporinus*, and *A. sagrei* as out-groups. Phylogenetic trees were constructed using Bayesian inference in the program BEAST version 1.5.3 (Drummond and Rambaut 2007). The program jModeltest version 0.1.1 (Posada 2008) was used to identify the best-fit model of nucleotide frequencies, substitution model, and transition-transversion ratio as evaluated by both Akaike Information Criterion (AIC) and Bayesian Information Criterion (BIC). The identified model, HKY + G, was then used to perform three independent runs of 20 million generations each. Analysis of the MCMC run via Tracer determined the burn-in period for the analyses to be 2.5 million generations. Twenty million generations ensured thorough sampling after the burn-in period.

Divergence times were estimated by applying a relaxed molecular clock model across the trees, using a rate of molecular evolution of 1.3% divergence/million years (0.65% change/lineage/million years) as in previous studies of reptiles using the ND2, tRNA^Trp^, and tRNA^Ala^ regions of the mitochondrial genome (Macey et al. 1998). This rate was used in previous phylogenetic analyses of the *carolinensis* group to date divergence times between species (Glor et al. 2004, 2005) and therefore, in the absence of informative fossils within the clade, we used this rate for comparisons with previous results.

Maximum Likelihood (ML) analyses were performed using the program RAxML version 7.3.0 (Stamatakis 2006; Stamatakis et al. 2008) implemented on the CIPRES cluster (Miller et al. 2010). We used 1000 iterations of the novel bootstrapping algorithm in RAxML to obtain branch supports. The rapid bootstrap analysis and search for the best-scoring ML tree was conducted in a single program run. A GTR + Optimization of substitution rates + GAMMA model of rate heterogeneity was used for bootstrap and final analysis. The alpha parameter was estimated by default in this option.

### Principal components analysis

To visualize population differentiation across the range of *A. carolinensis*, a principal components analysis (PCA) was conducted using SMARTPCA (Patterson et al. 2006) as implemented in Eigensoft version 3.0 (Price et al. 2006). Scores from the first three principal components were plotted using the program R (R Core Development Team 2010).

### Molecular diversity and gene flow

We used DNAsp version 5 (Librado and Rozas 2009) to calculate the number of haplotypes, haplotype diversity (Hd, Nei 1987), average number of nucleotide differences (K, Tajima 1983), and nucleotide diversity (π, Nei 1987). Gene flow was calculated by estimating the proportion of genetic diversity explained by allele frequency differences among populations (*F*_*ST*_, Lynch and Crease 1990), and corrected for multiple hits using the Jukes-Cantor distance model (*N*_*ST*_, Nei 1982) considering that the probability of multiple substitutions at a single site increases as two sequences diverge. In addition, these parameters were measured for each major clade identified from the phylogenetic analyses. A Wilcoxon rank sum test was used to test for significant differences in nucleotide diversity between clades.

### Demographic history

We calculated site frequency spectra to infer population size changes for the species and individual clades using the population size changes option in DNAsp version 5 (Librado and Rozas 2009). The observed distributions of allele frequencies were then compared with expected frequencies under a model of constant population size to determine possible shifts in the site frequency spectrum. Tajima's *D* was calculated to assess significance of the observed shifts in the frequency spectrum.

Under a demographic model of constant population size, the distribution of the count of alleles should decrease as the allele frequency increases and Tajima's *D* (Tajima 1989) should approximate zero under this scenario. In contrast, a population expansion event should be represented by an increase in the number of rare alleles, causing Tajima's *D* to become positive. A bottleneck event, however, should increase the proportion of medium to high frequency alleles, resulting in a negative Tajima's *D* statistic.

## Results

Bayesian and maximum likelihood analyses of mtDNA haplotypes recovered nearly identical topologies, including relationships among major clades of the *A. carolinensis*/*A. porcatus* species complex (for full Bayesian and ML trees see Files S1 and S2, respectively). A monophyletic *A. carolinensis* was well supported and most closely related to populations of *A. porcatus* from western Cuba (Fig. 1B). A relaxed molecular clock model places the divergence of *A. carolinensis* from *A. porcatus* pre-Pliocene, ∼6.8–17.8 MYA. All other *carolinensis* subgroup species were more closely related to East Cuban *A. porcauts. Anolis brunneus, A. longiceps*, and *A. maynardi* form a monophyletic group that diverged from East Cuban *A. porcatus* ∼11.5–23.9 MYA, whereas *A. smaragdinus* diverged from East Cuban *A. porcatus* ∼4.4–10.9 MYA.

Within the *A. carolinensis* clade, populations from the Central Florida Clade I including Highlands Hammock were basal. Divergence between central Florida populations and those from the rest of the range was estimated at ∼6.8–12.6 MYA. Within the Central Florida Clade I, the Highland Hammock population formed a strongly supported monophyletic group, whereas the other populations displayed some degree of paraphyly with respect to one another. The divergence time between Highland Hammock and the rest of the Central Florida Clade I was estimated at ∼3.1–8.1 MYA. Within the Southern Florida Clade, southeastern populations (Miami metropolitan area) formed a paraphyletic group with respect to the southwestern (Fort Myers) population. These two regions were separated from Central Florida Clade II, North Carolina, and Gulf Coast/Inland populations by ∼5.1–10.0 MYA. Populations across the majority of the species range from Texas to the central Atlantic coast formed a strongly supported, but shallowly divergent clade with a substantial degree of paraphyly among populations. This geographically widespread clade diverged from its most closely related populations (Orlando, FL, Palatka, FL, and North Carolina) ∼2.5–6.1 MYA. North Carolina samples formed a well-supported, monophyletic clade that was most closely related to populations from inland Florida (Central Florida Clade II). These populations were estimated to have separated ∼1.4–4.1 MYA.

**Figure 2:**
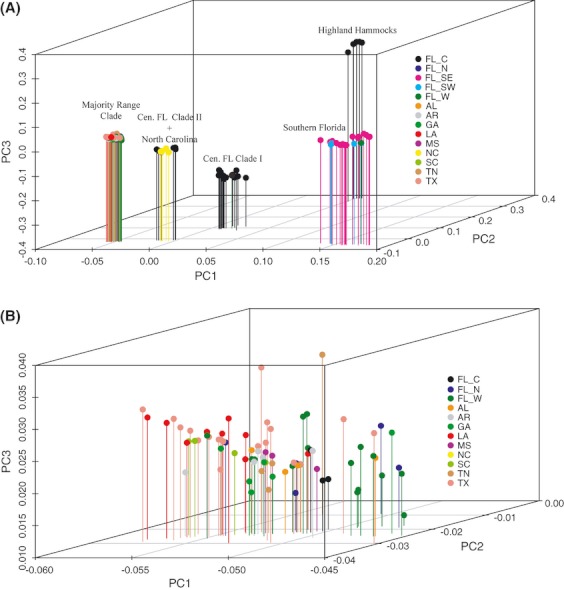
Principal components analysis of mitochondrial DNA sequences from populations across the range of *Anolis carolinensis*. (A) Genetic clustering for all samples in the dataset. (B) Genetic clustering for populations within the Gulf Coast/Inland Clade only. The *x*-axis represents principal component axis 1, the z-axis corresponds to principal component axis 2, and the *y*-axis corresponds to principal component axis 3. Major clades recovered from phylogenetic analyses are labeled above their corresponding clusters in principal component space. Each individual is represented by a pin and color coded by geographic region as shown in the figure legend.

The PCA showed five distinct population clusters (Fig. 2A) with the proximity of clusters closely reflecting the phylogenetic relationships recovered in the Bayesian and the maximum likelihood trees. Populations from southeastern and southwestern Florida clustered together. Central Florida populations split into three distinct clusters, one comprised of all individuals from Highland Hammock, another comprised of all other individuals from Central Florida Clade I, and the last combined Central Florida Clade II with the North Carolina populations. Populations within the Gulf Coast/Inland Clade formed a single cluster, but no discernible clustering can be seen within this clade (Fig. 2B).

Nucleotide diversity estimates within Florida clades were on average higher than in populations from other parts of the species' range. The diversity in the Gulf Coast/Inland Clade was significantly lower than in the Central Florida Clade II (W = 33, *P* = 0.04) and in southeast Florida (W = 64, *P* = 0.007), and was also lower than in Central Florida Clade I and southwest Florida, but not significantly so (W = 63, *P* = 0.12 and W = 16, *P* = 0.17, respectively). When populations from the Central Florida and Southern Florida Clades were combined, each group displayed significantly higher levels of variation than the Gulf Coast/Inland Clade (W = 23, *P* = 0.022 and W = 5, *P* = 0.003, respectively). Diversity in North Carolina did not differ from other clades; however, sampling in this region was limited. For a full table of diversity statistics see Table S2.

*F*_*ST*_ and *N*_*ST*_ statistics showed identical patterns of relationship between major clades of the species and therefore we report only the *F*_*ST*_ values. Within Florida, southeastern and southwestern populations showed the lowest levels of genetic differentiation (*F*_*ST*_ = 0.36), whereas the highest level of genetic differentiation was found between Central Florida Clade I and Southwest Florida (*F*_*ST*_ = 0.67). Across the entire range of *A. carolinensis*, North Carolina showed the highest levels of genetic differentiation as compared with other populations (see Table S3), with *F*_*ST*_ values ranging from 0.79 to 0.85 in all comparisons except with Central Florida Clade II (*F*_*ST*_ = 0.41). A similar pattern was found for the Gulf Coast/Inland Clade with a relatively high level of differentiation from all Florida regions (*F*_*ST*_ = 0.74–0.81), but lower differentiation with Central Florida Clade II (*F*_*ST*_ = 0.46). Within the Gulf Coast/Inland Clade there was no significant difference in genetic diversity among populations. For a full list of *F*_*ST*_ values, see Table S3.

**Figure 3:**
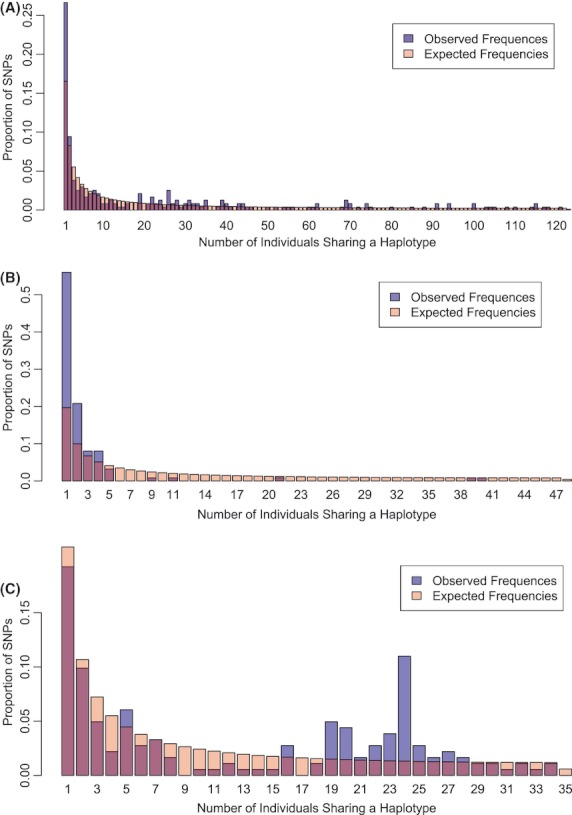
Site frequency spectra of haplotype alleles for geographic groups of *Anolis carolinensis*. (A) All populations collected across the range of the species. (B) Populations from Gulf Coast/Inland Clade identified in phylogenetic analyses. (C) Populations from Florida only. The *x*-axis of each graph represents the number of individuals that share a given haplotype and the *y*-axis represents the proportion of variable sites represented by each category. Observed spectra are colored blue and the expected distribution of alleles under a model of constant population size is colored translucent red. Therefore, areas of overlap between the observed and expected distributions appear purple.

The observed distribution of the frequency spectrum for all *A. carolinensis* populations showed a slightly higher proportion of rare alleles than expected under a model of constant population size (Fig. 3A). Furthermore, Tajima's *D* for the species was negative, as expected under population expansion. However, the deviation from neutral expectations was not significant (*D* = −0.8175, *P* > 0.10), providing weak evidence for population expansion when considering all populations. When analyzed separately, the Gulf Coast/Inland Clade populations showed a greater shift in the spectrum toward rare alleles (Fig. 3B) and Tajima's *D* for the Gulf Coast/Inland Clade was significantly negative (*D* = −2.56, *P* < 0.001), supporting population expansion within this clade. In contrast, the frequency spectrum for populations within Florida displayed an overrepresentation of intermediate frequency alleles when compared with the expected distribution (Fig. 3C), suggesting a recent population bottleneck. However, Tajima's *D* for these data was not significant (*D* = 0.064, *P* > 0.10).

## Discussion

Phylogeographic analyses reveal patterns of divergence within *A. carolinensis* similar to those observed in many other species of widespread anoles. There are five highly differentiated clades within the species; three of these clades occur within Florida, one in North Carolina, and one encompasses populations across the Gulf Coast, Atlantic Coast, and inland southeastern United States. (Fig. 1). The geographic boundaries between these clades do not correspond to major river boundaries of the region described in previous phylogeographic studies (reviewed in Soltis et al. 2006). However, the Appalachian Mountains may serve as a barrier to migration between the Gulf Coast/Inland Clade and North Carolina Clade at the northern edge of the species' range.

Phylogenetic relationships support previous hypotheses for the geographic origin of *A. carolinensis*. Williams (1969, 1989) identified Cuba as the likely point of origin of *A. carolinensis* and other West Indian members of the *carolinensis* subgroup, and Buth et al. (1980) suggested a Pliocene colonization of *A. carolinensis* to the United States. Glor et al. (2005) corroborated both of these hypotheses with a phylogeographic analysis, dating diversification of the *carolinensis* subgroup out of Cuba in the late Miocene or early Pliocene. The monophyly of *A. carolinensis* with respect to its ancestral congener supports the results of Glor et al. (2005), suggesting a single colonization event to southeastern United States from western Cuba. However, divergence times suggest that diversification of *carolinensis* subgroup species from ancestral populations in Eastern Cuba may have occurred much earlier than reported in Glor et al. (2005). Whereas *A. smaragdinus* diverged from East Cuban *A. porcatus* sometime within the Miocene to Pliocene (∼3.5–16.2 MYA) based on our estimates, the divergence event that separates the clade containing *A. brunneus, A. longiceps*, and *A. maynardi* is estimated at ∼9.3–32.6 MYA, perhaps the early Oligocene. However, because our dataset only represents a portion of the mitochondrial region used by Macey et al. (1998), including a higher proportion of protein coding sequence with respect to tRNAs and not including the slower evolving COI gene, the rate of evolution within this region may be faster and divergence times may be younger than our estimates.

Based on Bayesian and maximum likelihood tree topologies, central Florida, a previously described refugial area (Scott and Upchurch 1982; Riggs 1984; Hayes and Harrison 1992; Ellsworth et al. 1994), holds the ancestral populations of the species. Branching times and geographic positioning of the Gulf Coast/Inland, North Carolina, and Southern Florida Clades suggests multiple migrations out of Florida into the species current range. Divergence times place these migration events between the mid-Miocene to the early Pleistocene.

North Carolina populations may represent a Pleistocene refugial population. Divergence times place the separation of North Carolina and Central Florida Clade II populations within the Gelasian and Calabrian stages of the Pleistocene, ∼0.78–2.5 MYA. This region has also been identified as a possible refugium for other species (Brant and Orti 2003; Church et al. 2003; Austin et al. 2004), and displays a high degree of genetic differentiation from geographically proximate populations in our analysis. Alternatively, the geographic separation of this haplogroup from central Florida could signify mitochondrial introgression of the expanding Gulf Coast/Inland Clade populations into the Atlantic Coast.

Most of the species' range shows little phylogeographic structure (i.e., the Gulf Coast/Inland Clade), indicating a possible recent range expansion across this region. This hypothesis is further supported by the observed shift in the site frequency spectrum toward rare alleles and a significantly negative Tajima's *D* statistic. Earliest divergence times within this clade place the start of this expansion within the Pleistocene, ∼0.96–2.0 MYA. In addition, the short branch lengths and extensive paraphyly that characterize the Gulf Coast/Inland Clade may explain previous observations of close relationships for some populations across this region.

Webster et al. (1972) compared the genetic distance between populations of *A. carolinensis* from Florida, Louisiana, and Texas, reporting a high degree of genetic uniformity among these populations using allozymes, with Florida and Louisiana populations more closely related to each other than to Texas. These results contrast with the findings of this study, which show that Florida populations are highly diverged from Louisiana and Texas. The Silver Springs Florida population used by Webster et al. (1972) is near the border of the Gulf Coast/Inland Clade and Central Florida Clade I haplogroups identified in this study. The observed similarity in allozyme frequencies may be a result of nuclear gene flow between these two groups.

Buth et al. (1980) used electrophoretic comparisons at 35 allozyme loci to estimate genetic differences between Texas and Georgia populations of *A. carolinensis* and western Cuba populations of *A. porcatus*. They found small genetic distances separated Texas and Georgia, a result consistent with our findings.

Wade et al. (1983) found that populations from the Gulf Coast (Birmingham and Auburn, AL, and Tellico, TN) and Gainesville, FL, were genetically similar based on allozyme data. This again, may reflect nuclear genetic admixture between Gulf Coast/Inland Clade and Central Florida Clade haplogroups. San Marcos, TX, displayed intermediate genetic distance between this group and New Orleans, LA. Populations from Naples, FL, which have been suggested as a subspecies based on morphological (Christman 1980) and physiological (Wilson and Echternacht 1990) evidence, are the most genetically distinct group. This agrees with our finding that southern Florida populations represent a genetically distinct lineage within the species, although the geographic range described by these studies is much smaller than that occupied by the Southern Florida haplogroup.

A recent expansion of populations within the Gulf Coast/Inland Clade explains the small genetic distances reported in earlier studies. The Lower Mississippi Valley, a hypothesized refugial area (Davis 1981; Delcourt and Delcourt 1981) lies in the center of the Gulf Coast/Inland Clade haplotype distribution. The expansion of this clade may have occurred out of this region; however, further data are needed to test this hypothesis.

The observed shifts in the site frequency spectrum of the ND2 region of *A. carolinensis* show signatures of population expansion by the species. However, the drastic shift seen in the Gulf Coast/Inland populations suggests that the species-level signature of a shift toward rare alleles is being driven by this clade. In the absence of this clade, *A. carolinensis* populations display shifts, although statistically nonsignificant, in site frequency spectra expected from a bottleneck event. Given the topology of the tree, these data may highlight molecular signatures of both the bottleneck associated with the initial colonization of the species from Cuba into mainland Florida and the recent expansion of populations out of Florida into its current range. To better understand the genetic effects of this demographic history, a multilocus study including nuclear markers is needed.

Clustering patterns identified in the PCA largely mirror the topological structure recovered in the Bayesian and maximum likelihood trees. The tight clustering pattern of most major clades reflects a high degree of genetic structure. Conversely, the lack of clustering of populations within the Gulf Coast/Inland Clade further supports the hypothesis of low genetic differentiation between populations occupying most of the species' range, likely due to a recent expansion event.

## Conclusion

Pleistocene glaciation has influenced the current distribution and population relatedness within and between species of *Anolis* lizards and many other species, and has been proposed as a major factor for Caribbean *Anolis* diversification (Glor et al. 2004). The timing and pattern of divergence events suggest that cyclical variation in temperature throughout southeastern United States may have had a significant effect on population expansion across the majority of the *A. carolinensis* range. Molecular analyses suggest there are at least five highly divergent lineages within *A. carolinensis*. However, despite the Miocene-Pliocene arrival of the species to the southeastern United States, three of these haplogroups represent limited geographic distributions within Florida, the likely region of colonization. It is not until the periodic warming cycles of the Pleistocene that significant changes in the geographic distribution occur, leading to migrations north along the Atlantic coast, west across the Gulf coast and inland southeastern United States, and establishment of populations in newly habitable areas.

Our work sets the stage for future studies of phenotypic and genetic variation, demographic history, and adaptation within *A. carolinensis*. Future studies of the nuclear genome of this species will elucidate the importance of demographic history in shaping patterns of variation and adaptation. We are aware of the limitations of phylogeographic studies based solely on mitochondrial loci (Edwards and Bensch 2009). Therefore, we encourage additional study of phylogeographic patterns using nuclear markers.

## Supporting Information

**File S1.** Results of the Bayesian phylogenetic analysis of *Anolis carolinensis* and *A. porcatus* mtDNA sequences.

**File S2.** Results of the maximum likelihood phylogenetic analysis of *Anolis carolinensis* and *A. porcatus* mtDNA sequences.

**Table S1.** Sampling localities and Genbank numbers for mtDNA haplotypes used in this study, including previously published data from Glor et al. 2004 and Kolbe et al. 2007 and new sequence data from this study.

**Table S2.** Diversity statistics for *Anolis carolinensis* populations across the species range. N, number of individuals collected for each populations; S, the number of segregating sites in each population; h, the number of haplotypes in each population; Hd, haplotype diversity in each population; K, the average number of differences in each populationl; Pi, nucleotide diversity in each population; PiJC, nucleotide diversity in each population with Jukes Cantor correction.

**Table S3.** Pairwise genetic differentiation between populations across the native range of *Anolis carolinensis* calculated using the standard fixation index, *F_ST_*, and the fixation index corrected for multiple hits using the Jukes–Cantor distance model, *N_ST_*.
