# Supplementary material for: Out of Florida: mtDNA reveals patterns of migration and Pleistocene range expansion of the Green Anole lizard (Anolis carolinensis)
Source: Ecol Evol. 2012 Aug 8;2(9):2274–84. doi: 10.1002/ece3.324 (PMC3488677; doi:10.1002/ece3.324)
Supplement: Supplementary file 4 [file ece30002-2274-SD4.pdf]

	N	S	h	Hd	K	Pi	PiJC
AR	5	2	3	0.7	0.8	0.00171	0.00171
MS	5	2	3	0.8	1	0.00213	0.00214
AL	5	2	3	0.8	1	0.00213	0.00214
Chipola_RiverFL	8	4	5	0.78571	1	0.00213	0.00214
NC	6	3	4	0.8667	1.2	0.00256	0.00256
CC_TX	5	3	3	0.7	1.2	0.00256	0.00257
GainesvilleFL	3	2	3	1	1.3333	0.00284	0.00285
SC	5	3	4	0.9	1.4	0.00299	0.00299
OR_TX	5	3	3	0.7	1.4	0.00299	0.00299
GA	5	3	3	0.8	1.4	0.00299	0.00299
TY_TX	5	3	4	0.9	1.4	0.00299	0.00299
Houston_TX	4	3	3	0.83333	1.5	0.0032	0.00321
Highland_HammocksFL	5	5	4	0.9	2	0.00426	0.00428
S_LA	5	4	3	0.8	2.2	0.00469	0.00471
BV_TX	5	5	4	0.9	2.2	0.00469	0.00471
N_FL	5	6	4	0.9	2.6	0.00554	0.00557
E_TN	5	5	3	0.7	2.6	0.00554	0.00558
W_TN	5	7	4	0.9	2.8	0.00597	0.00601
InvernessFL	3	6	3	1	4	0.00853	0.00859
N_LA	5	10	4	0.9	4.6	0.00981	0.00989
SE_FL	5	13	5	1	5.8	0.01237	0.01248
SW_FL	4	13	4	1	7.33333	0.01564	0.01582
RedRoadFL	8	26	8	1	8.32143	0.01774	0.01802
Floral_CityFL	4	17	4	1	8.5	0.01812	0.01837
PalatkaFL	3	13	3	1	8.66667	0.01848	0.01876
Doc_ThomasFL	5	18	5	1	9	0.01919	0.01947
ParklandFL	3	14	3	1	9.33333	0.0199	0.0217
W-FL	6	29	3	0.6	9.876	0.02104	0.02193
C_FL	5	39	5	1	17.4	0.0371	0.03867
EconfinaFL	4	32	3	0.83333	21.16667	0.04513	0.04727
