# Supplementary figures and images for: Out of Florida: mtDNA reveals patterns of migration and Pleistocene range expansion of the Green Anole lizard (Anolis carolinensis)
Source: Ecol Evol. 2012 Aug 8;2(9):2274–84. doi: 10.1002/ece3.324 (PMC3488677; doi:10.1002/ece3.324)

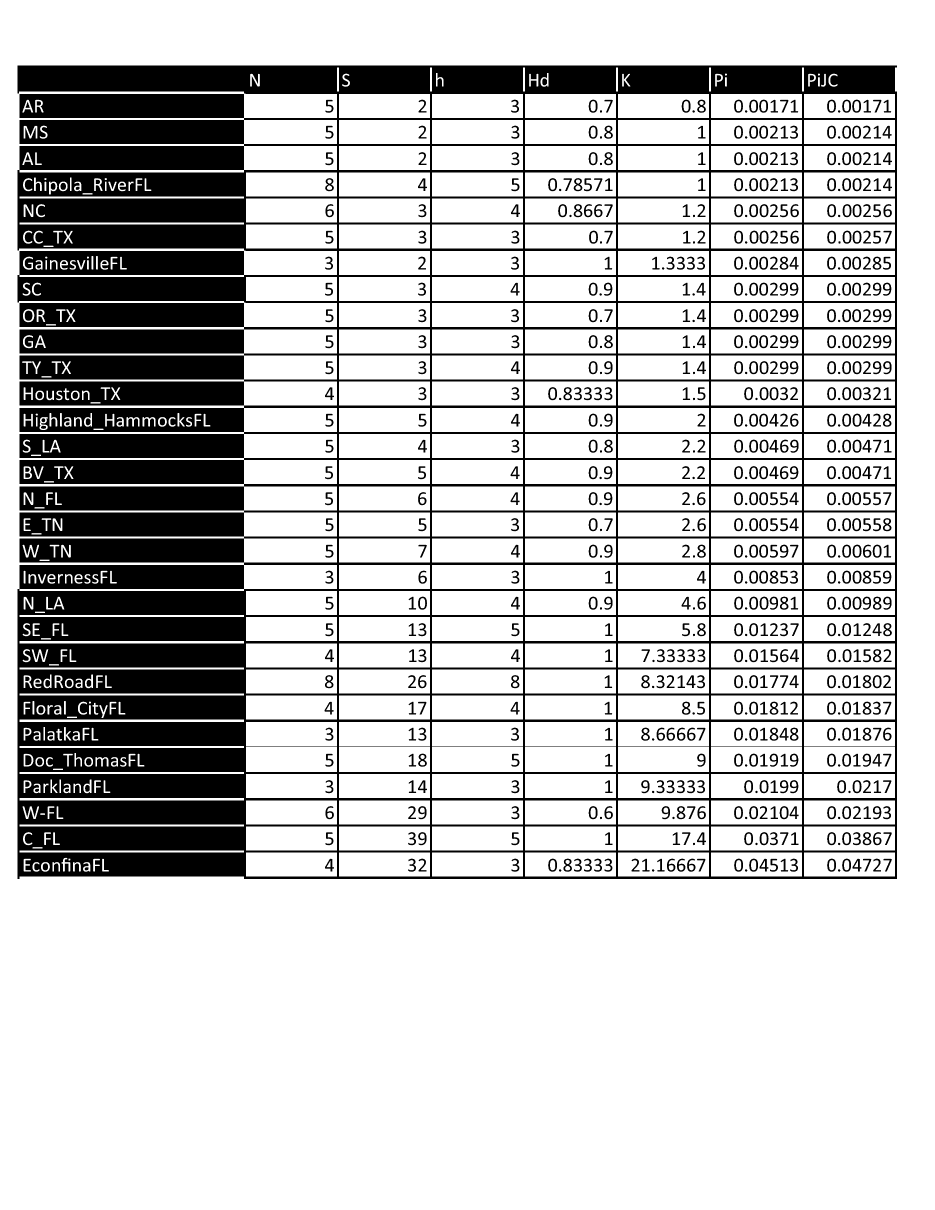

Supplement: Supplementary file 5 [file ece30002-2274-SD6.png]

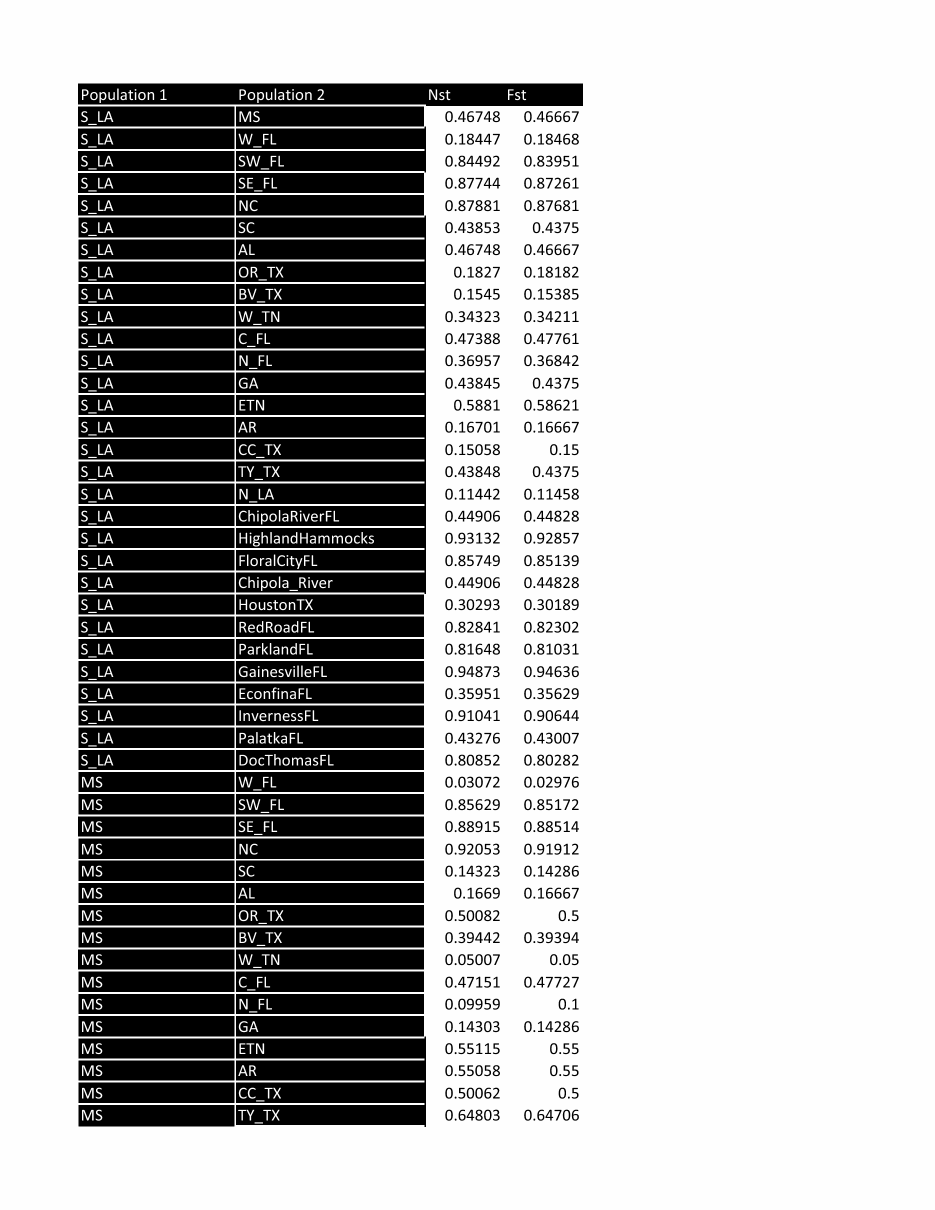

Supplement: Supplementary file 8 [file ece30002-2274-SD7.png]
