# Supplementary material for: Out of Florida: mtDNA reveals patterns of migration and Pleistocene range expansion of the Green Anole lizard (Anolis carolinensis)
Source: Ecol Evol. 2012 Aug 8;2(9):2274–84. doi: 10.1002/ece3.324 (PMC3488677; doi:10.1002/ece3.324)
Supplement: Supplementary file 7 [file ece30002-2274-SD5.pdf]

Population 1	Population 2	Nst	Fst
S_LA	MS	0.46748	0.46667
S_LA	W_FL	0.18447	0.18468
S_LA	SW_FL	0.84492	0.83951
S_LA	SE_FL	0.87744	0.87261
S_LA	NC	0.87881	0.87681
S_LA	SC	0.43853	0.4375
S_LA	AL	0.46748	0.46667
S_LA	OR_TX	0.1827	0.18182
S_LA	BV_TX	0.1545	0.15385
S_LA	W_TN	0.34323	0.34211
S_LA	C_FL	0.47388	0.47761
S_LA	N_FL	0.36957	0.36842
S_LA	GA	0.43845	0.4375
S_LA	ETN	0.5881	0.58621
S_LA	AR	0.16701	0.16667
S_LA	CC_TX	0.15058	0.15
S_LA	TY_TX	0.43848	0.4375
S_LA	N_LA	0.11442	0.11458
S_LA	ChipolaRiverFL	0.44906	0.44828
S_LA	HighlandHammocks	0.93132	0.92857
S_LA	FloralCityFL	0.85749	0.85139
S_LA	Chipola_River	0.44906	0.44828
S_LA	HoustonTX	0.30293	0.30189
S_LA	RedRoadFL	0.82841	0.82302
S_LA	ParklandFL	0.81648	0.81031
S_LA	GainesvilleFL	0.94873	0.94636
S_LA	EconfinaFL	0.35951	0.35629
S_LA	InvernessFL	0.91041	0.90644
S_LA	PalatkaFL	0.43276	0.43007
S_LA	DocThomasFL	0.80852	0.80282
MS	W_FL	0.03072	0.02976
MS	SW_FL	0.85629	0.85172
MS	SE_FL	0.88915	0.88514
MS	NC	0.92053	0.91912
MS	SC	0.14323	0.14286
MS	AL	0.1669	0.16667
MS	OR_TX	0.50082	0.5
MS	BV_TX	0.39442	0.39394
MS	W_TN	0.05007	0.05
MS	C_FL	0.47151	0.47727
MS	N_FL	0.09959	0.1
MS	GA	0.14303	0.14286
MS	ETN	0.55115	0.55
MS	AR	0.55058	0.55
MS	CC_TX	0.50062	0.5
MS	TY_TX	0.64803	0.64706

MS	N_LA	0.17532	0.17647
MS	ChipolaRiverFL	0.09108	0.09091
MS	HighlandHammocks	0.94765	0.94565
MS	FloralCityFL	0.86797	0.86272
MS	Chipola_River	0.09108	0.09091
MS	HoustonTX	0.56236	0.5614
MS	RedRoadFL	0.8379	0.8334
MS	ParklandFL	0.82462	0.81935
MS	GainesvilleFL	0.9643	0.96269
MS	EconfinaFL	0.32375	0.32212
MS	InvernessFL	0.92407	0.92089
MS	PalatkaFL	0.41724	0.41532
MS	DocThomasFL	0.81682	0.81203
W_FL	SW_FL	0.63795	0.63404
W_FL	SE_FL	0.70875	0.70551
W_FL	NC	0.64937	0.65296
W_FL	SC	0.01832	0.01744
W_FL	AL	0.03072	0.02976
W_FL	OR_TX	0.17057	0.17157
W_FL	BV_TX	0.14521	0.14623
W_FL	W_TN	0.00249	0
W_FL	C_FL	0.30007	0.3056
W_FL	N_FL	0.02847	0.02604
W_FL	GA	0.01827	0.01744
W_FL	ETN	0.25762	0.25794
W_FL	AR	0.16483	0.16667
W_FL	CC_TX	0.16042	0.16162
W_FL	TY_TX	0.27559	0.27778
W_FL	N_LA	0.0669	0.06466
W_FL	ChipolaRiverFL	0.00558	0.00458
W_FL	HighlandHammocks	0.78461	0.78346
W_FL	FloralCityFL	0.733	0.72723
W_FL	Chipola_River	0.00558	0.00458
W_FL	HoustonTX	0.17456	0.17831
W_FL	RedRoadFL	0.63361	0.63039
W_FL	ParklandFL	0.62768	0.62353
W_FL	GainesvilleFL	0.81791	0.81639
W_FL	EconfinaFL	0.1467	0.14587
W_FL	InvernessFL	0.77747	0.77432
W_FL	PalatkaFL	0.16457	0.166
W_FL	DocThomasFL	0.60221	0.59915
SW_FL	SE_FL	0.61219	0.60679
SW_FL	NC	0.85048	0.84578
SW_FL	SC	0.84831	0.84349
SW_FL	AL	0.85629	0.85172
SW_FL	OR_TX	0.85586	0.85097
SW_FL	BV_TX	0.84042	0.83506

SW_FL	W_TN	0.82785	0.82222
SW_FL	C_FL	0.60919	0.60363
SW_FL	N_FL	0.83376	0.82814
SW_FL	GA	0.8483	0.84349
SW_FL	ETN	0.84497	0.83927
SW_FL	AR	0.86376	0.85928
SW_FL	CC_TX	0.85813	0.85338
SW_FL	TY_TX	0.86083	0.85589
SW_FL	N_LA	0.80715	0.80045
SW_FL	ChipolaRiverFL	0.8544	0.84985
SW_FL	HighlandHammocks	0.85577	0.85043
SW_FL	FloralCityFL	0.76587	0.75734
SW_FL	Chipola_River	0.8544	0.84985
SW_FL	HoustonTX	0.84642	0.84155
SW_FL	RedRoadFL	0.35607	0.35277
SW_FL	ParklandFL	0.43017	0.42529
SW_FL	GainesvilleFL	0.86436	0.85946
SW_FL	EconfinaFL	0.52432	0.52101
SW_FL	InvernessFL	0.81857	0.81215
SW_FL	PalatkaFL	0.7096	0.70186
SW_FL	DocThomasFL	0.36102	0.35696
SE_FL	NC	0.89369	0.88947
SE_FL	SC	0.88182	0.87755
SE_FL	AL	0.88756	0.88356
SE_FL	OR_TX	0.88743	0.88312
SE_FL	BV_TX	0.87321	0.86842
SE_FL	W_TN	0.86328	0.85818
SE_FL	C_FL	0.66888	0.66279
SE_FL	N_FL	0.8669	0.86184
SE_FL	GA	0.87977	0.87552
SE_FL	ETN	0.87298	0.86792
SE_FL	AR	0.89535	0.89145
SE_FL	CC_TX	0.88979	0.88562
SE_FL	TY_TX	0.89114	0.88679
SE_FL	N_LA	0.83747	0.83139
SE_FL	ChipolaRiverFL	0.88756	0.88356
SE_FL	HighlandHammocks	0.88512	0.88037
SE_FL	FloralCityFL	0.80321	0.79513
SE_FL	Chipola_River	0.88756	0.88356
SE_FL	HoustonTX	0.88081	0.87648
SE_FL	RedRoadFL	0.44857	0.44513
SE_FL	ParklandFL	0.01346	0.01304
SE_FL	GainesvilleFL	0.89583	0.89148
SE_FL	EconfinaFL	0.5735	0.56991
SE_FL	InvernessFL	0.85957	0.85359
SE_FL	PalatkaFL	0.76638	0.75889
SE_FL	DocThomasFL	0.31493	0.31227

NC	SC	0.90744	0.9058
NC	AL	0.92053	0.91912
NC	OR_TX	0.91382	0.91216
NC	BV_TX	0.89021	0.88816
NC	W_TN	0.85929	0.85714
NC	C_FL	0.44923	0.45294
NC	N_FL	0.87032	0.86806
NC	GA	0.9046	0.90299
NC	ETN	0.88639	0.88415
NC	AR	0.93185	0.93056
NC	CC_TX	0.91933	0.91781
NC	TY_TX	0.9194	0.91772
NC	N_LA	0.81455	0.81169
NC	ChipolaRiverFL	0.91839	0.91698
NC	HighlandHammocks	0.95593	0.95376
NC	FloralCityFL	0.87352	0.86803
NC	Chipola_River	0.91839	0.91698
NC	HoustonTX	0.91319	0.91148
NC	RedRoadFL	0.83938	0.83465
NC	ParklandFL	0.83383	0.82826
NC	GainesvilleFL	0.96413	0.96238
NC	EconfinaFL	0.52845	0.52912
NC	InvernessFL	0.92689	0.92353
NC	PalatkaFL	0.38353	0.38333
NC	DocThomasFL	0.82971	0.82454
SC	AL	0.14323	0.14286
SC	OR_TX	0.3866	0.38596
SC	BV_TX	0.38419	0.38356
SC	W_TN	0.04587	0.04545
SC	C_FL	0.4641	0.46953
SC	N_FL	0.09086	0.09091
SC	GA	0.12538	0.125
SC	ETN	0.5252	0.52381
SC	AR	0.50066	0.5
SC	CC_TX	0.45909	0.45833
SC	TY_TX	0.61223	0.61111
SC	N_LA	0.16597	0.16667
SC	ChipolaRiverFL	0.07725	0.07692
SC	HighlandHammocks	0.94021	0.93796
SC	FloralCityFL	0.86161	0.8561
SC	Chipola_River	0.07725	0.07692
SC	HoustonTX	0.5257	0.52459
SC	RedRoadFL	0.83163	0.82687
SC	ParklandFL	0.81655	0.81103
SC	GainesvilleFL	0.95788	0.95601
SC	EconfinaFL	0.31164	0.30989
SC	InvernessFL	0.91746	0.91401

SC	PalatkaFL	0.40782	0.40551
SC	DocThomasFL	0.80927	0.80422
AL	OR_TX	0.50082	0.5
AL	BV_TX	0.42912	0.42857
AL	W_TN	0.05007	0.05
AL	C_FL	0.47151	0.47727
AL	N_FL	0.09959	0.1
AL	GA	0.14303	0.14286
AL	ETN	0.53715	0.53608
AL	AR	0.55058	0.55
AL	CC_TX	0.50062	0.5
AL	TY_TX	0.64803	0.64706
AL	N_LA	0.17532	0.17647
AL	ChipolaRiverFL	0.09108	0.09091
AL	HighlandHammocks	0.94765	0.94565
AL	FloralCityFL	0.86634	0.86111
AL	Chipola_River	0.09108	0.09091
AL	HoustonTX	0.56236	0.5614
AL	RedRoadFL	0.83545	0.83098
AL	ParklandFL	0.82203	0.81678
AL	GainesvilleFL	0.96381	0.9622
AL	EconfinaFL	0.31497	0.31373
AL	InvernessFL	0.92305	0.91987
AL	PalatkaFL	0.41724	0.41532
AL	DocThomasFL	0.81392	0.80916
OR_TX	BV_TX	0.10038	0.1
OR_TX	W_TN	0.34452	0.34375
OR_TX	C_FL	0.4957	0.5
OR_TX	N_FL	0.37583	0.375
OR_TX	GA	0.4624	0.46154
OR_TX	ETN	0.61696	0.61538
OR_TX	AR	0.08395	0.08333
OR_TX	CC_TX	0.07215	0.07143
OR_TX	TY_TX	0.46244	0.46154
OR_TX	N_LA	0.1173	0.11765
OR_TX	ChipolaRiverFL	0.47906	0.47826
OR_TX	HighlandHammocks	0.94324	0.94097
OR_TX	FloralCityFL	0.86731	0.86173
OR_TX	Chipola_River	0.47906	0.47826
OR_TX	HoustonTX	0.29374	0.29268
OR_TX	RedRoadFL	0.83825	0.83339
OR_TX	ParklandFL	0.82554	0.81991
OR_TX	GainesvilleFL	0.95978	0.95791
OR_TX	EconfinaFL	0.35932	0.35708
OR_TX	InvernessFL	0.92115	0.91768
OR_TX	PalatkaFL	0.47089	0.46831
OR_TX	DocThomasFL	0.81812	0.81295

BV_TX	W_TN	0.30642	0.30556
BV_TX	C_FL	0.4827	0.48637
BV_TX	N_FL	0.33419	0.33333
BV_TX	GA	0.40067	0.4
BV_TX	ETN	0.57322	0.57143
BV_TX	AR	0.06216	0.0625
BV_TX	CC_TX	0.05553	0.05556
BV_TX	TY_TX	0.4007	0.4
BV_TX	N_LA	0.10525	0.10526
BV_TX	ChipolaRiverFL	0.4079	0.40741
BV_TX	HighlandHammocks	0.92983	0.92708
BV_TX	FloralCityFL	0.85493	0.84887
BV_TX	Chipola_River	0.4079	0.40741
BV_TX	HoustonTX	0.24558	0.2449
BV_TX	RedRoadFL	0.82439	0.81906
BV_TX	ParklandFL	0.80994	0.80385
BV_TX	GainesvilleFL	0.94727	0.94491
BV_TX	EconfinaFL	0.3367	0.33428
BV_TX	InvernessFL	0.90828	0.90432
BV_TX	PalatkaFL	0.45246	0.44932
BV_TX	DocThomasFL	0.80243	0.79681
W_TN	C_FL	0.43201	0.43638
W_TN	N_FL	0.02193	0.02174
W_TN	GA	0.0093	0.00943
W_TN	ETN	0.26674	0.2663
W_TN	AR	0.35735	0.35714
W_TN	CC_TX	0.33382	0.33333
W_TN	TY_TX	0.501	0.5
W_TN	N_LA	0.11921	0.11905
W_TN	ChipolaRiverFL	0.00006	0
W_TN	HighlandHammocks	0.91768	0.91465
W_TN	FloralCityFL	0.84159	0.83528
W_TN	Chipola_River	0.00006	0
W_TN	HoustonTX	0.41194	0.41096
W_TN	RedRoadFL	0.80994	0.80437
W_TN	ParklandFL	0.79909	0.79271
W_TN	GainesvilleFL	0.9357	0.93305
W_TN	EconfinaFL	0.27847	0.27593
W_TN	InvernessFL	0.89526	0.89103
W_TN	PalatkaFL	0.35137	0.34848
W_TN	DocThomasFL	0.78929	0.78341
C_FL	N_FL	0.43881	0.44321
C_FL	GA	0.45006	0.45602
C_FL	ETN	0.49548	0.49799
C_FL	AR	0.50033	0.50543
C_FL	CC_TX	0.48624	0.49125
C_FL	TY_TX	0.52079	0.52429

C_FL	N_LA	0.42604	0.42827
C_FL	ChipolaRiverFL	0.46211	0.46821
C_FL	HighlandHammocks	0.6921	0.6891
C_FL	FloralCityFL	0.66175	0.65328
C_FL	Chipola_River	0.46211	0.46821
C_FL	HoustonTX	0.50527	0.50909
C_FL	RedRoadFL	0.60205	0.59621
C_FL	ParklandFL	0.60119	0.59413
C_FL	GainesvilleFL	0.72361	0.72012
C_FL	EconfinaFL	0.24835	0.24821
C_FL	InvernessFL	0.68854	0.68343
C_FL	PalatkaFL	0.10734	0.11136
C_FL	DocThomasFL	0.59329	0.58698
N_FL	GA	0.09073	0.09091
N_FL	ETN	0.44126	0.43966
N_FL	AR	0.39319	0.39286
N_FL	CC_TX	0.34976	0.34932
N_FL	TY_TX	0.525	0.52381
N_FL	N_LA	0.14278	0.14286
N_FL	ChipolaRiverFL	0.05208	0.05263
N_FL	HighlandHammocks	0.92086	0.91786
N_FL	FloralCityFL	0.84865	0.84233
N_FL	Chipola_River	0.05208	0.05263
N_FL	HoustonTX	0.43946	0.43836
N_FL	RedRoadFL	0.81546	0.8099
N_FL	ParklandFL	0.80246	0.79613
N_FL	GainesvilleFL	0.94092	0.93828
N_FL	EconfinaFL	0.3064	0.30303
N_FL	InvernessFL	0.90173	0.89752
N_FL	PalatkaFL	0.38187	0.37868
N_FL	DocThomasFL	0.79355	0.7877
GA	ETN	0.52514	0.52381
GA	AR	0.50053	0.5
GA	CC_TX	0.45897	0.45833
GA	TY_TX	0.61215	0.61111
GA	N_LA	0.12676	0.12791
GA	ChipolaRiverFL	0.07703	0.07692
GA	HighlandHammocks	0.94065	0.93841
GA	FloralCityFL	0.85857	0.85312
GA	Chipola_River	0.07703	0.07692
GA	HoustonTX	0.52561	0.52459
GA	RedRoadFL	0.8248	0.82014
GA	ParklandFL	0.81472	0.80924
GA	GainesvilleFL	0.95671	0.95485
GA	EconfinaFL	0.2935	0.29258
GA	InvernessFL	0.91596	0.91253
GA	PalatkaFL	0.38815	0.38618

GA	DocThomasFL	0.80373	0.79876
ETN	AR	0.64701	0.64583
ETN	CC_TX	0.62139	0.62
ETN	TY_TX	0.67911	0.67742
ETN	N_LA	0.42104	0.41935
ETN	ChipolaRiverFL	0.53959	0.53846
ETN	HighlandHammocks	0.92732	0.92434
ETN	FloralCityFL	0.8486	0.84233
ETN	Chipola_River	0.53959	0.53846
ETN	HoustonTX	0.63886	0.63717
ETN	RedRoadFL	0.82467	0.81903
ETN	ParklandFL	0.81269	0.80628
ETN	GainesvilleFL	0.94086	0.93828
ETN	EconfinaFL	0.34343	0.34164
ETN	InvernessFL	0.90168	0.89752
ETN	PalatkaFL	0.49473	0.49096
ETN	DocThomasFL	0.80456	0.79861
AR	CC_TX	0.00026	0
AR	TY_TX	0.5006	0.5
AR	N_LA	0.09875	0.1
AR	ChipolaRiverFL	0.52686	0.52632
AR	HighlandHammocks	0.95257	0.9507
AR	FloralCityFL	0.8738	0.86864
AR	Chipola_River	0.52686	0.52632
AR	HoustonTX	0.30373	0.30303
AR	RedRoadFL	0.84594	0.8415
AR	ParklandFL	0.83287	0.82766
AR	GainesvilleFL	0.96821	0.96674
AR	EconfinaFL	0.36101	0.35957
AR	InvernessFL	0.92899	0.92593
AR	PalatkaFL	0.47991	0.47794
AR	DocThomasFL	0.8259	0.82117
CC_TX	TY_TX	0.45902	0.45833
CC_TX	N_LA	0.09297	0.09375
CC_TX	ChipolaRiverFL	0.47678	0.47619
CC_TX	HighlandHammocks	0.94539	0.94326
CC_TX	FloralCityFL	0.86919	0.86376
CC_TX	Chipola_River	0.47678	0.47619
CC_TX	HoustonTX	0.27108	0.27027
CC_TX	RedRoadFL	0.84011	0.83541
CC_TX	ParklandFL	0.82673	0.82127
CC_TX	GainesvilleFL	0.96248	0.96074
CC_TX	EconfinaFL	0.35738	0.35543
CC_TX	InvernessFL	0.92357	0.92025
CC_TX	PalatkaFL	0.46996	0.46763
CC_TX	DocThomasFL	0.81968	0.81468
TY_TX	N_LA	0.31849	0.31818

TY_TX	ChipolaRiverFL	0.63731	0.63636
TY_TX	HighlandHammocks	0.94485	0.94257
TY_TX	FloralCityFL	0.87038	0.86475
TY_TX	Chipola_River	0.63731	0.63636
TY_TX	HoustonTX	0.52566	0.52459
TY_TX	RedRoadFL	0.84359	0.83865
TY_TX	ParklandFL	0.83147	0.82576
TY_TX	GainesvilleFL	0.9608	0.95892
TY_TX	EconfinaFL	0.39062	0.38844
TY_TX	InvernessFL	0.92345	0.91996
TY_TX	PalatkaFL	0.5219	0.51911
TY_TX	DocThomasFL	0.8247	0.81944
N_LA	ChipolaRiverFL	0.1236	0.125
N_LA	HighlandHammocks	0.89329	0.88926
N_LA	FloralCityFL	0.82703	0.81956
N_LA	Chipola_River	0.1236	0.125
N_LA	HoustonTX	0.20791	0.20779
N_LA	RedRoadFL	0.78667	0.78006
N_LA	ParklandFL	0.77622	0.76881
N_LA	GainesvilleFL	0.91397	0.91028
N_LA	EconfinaFL	0.30306	0.29791
N_LA	InvernessFL	0.87708	0.87177
N_LA	PalatkaFL	0.37047	0.36624
N_LA	DocThomasFL	0.76474	0.75783
ChipolaRiverFL	HighlandHammocks	0.94685	0.94485
ChipolaRiverFL	FloralCityFL	0.86603	0.86081
ChipolaRiverFL	Chipola_River	-0.14286	-0.14286
ChipolaRiverFL	HoustonTX	0.54639	0.54545
ChipolaRiverFL	RedRoadFL	0.83576	0.83129
ChipolaRiverFL	ParklandFL	0.82235	0.81711
ChipolaRiverFL	GainesvilleFL	0.96387	0.96226
ChipolaRiverFL	EconfinaFL	0.30842	0.30729
ChipolaRiverFL	InvernessFL	0.92318	0.92
ChipolaRiverFL	PalatkaFL	0.40387	0.40206
ChipolaRiverFL	DocThomasFL	0.81428	0.80952
HighlandHammocks	FloralCityFL	0.83578	0.8301
HighlandHammocks	Chipola_River	0.94685	0.94485
HighlandHammocks	HoustonTX	0.9425	0.94017
HighlandHammocks	RedRoadFL	0.84251	0.83707
HighlandHammocks	ParklandFL	0.82211	0.81602
HighlandHammocks	GainesvilleFL	0.94064	0.93842
HighlandHammocks	EconfinaFL	0.57709	0.57802
HighlandHammocks	InvernessFL	0.89477	0.89104
HighlandHammocks	PalatkaFL	0.82363	0.81818
HighlandHammocks	DocThomasFL	0.82768	0.82189
FloralCityFL	Chipola_River	0.86603	0.86081
FloralCityFL	HoustonTX	0.86578	0.86014

FloralCityFL	RedRoadFL	0.74149	0.73299
FloralCityFL	ParklandFL	0.74331	0.73383
FloralCityFL	GainesvilleFL	0.14246	0.14493
FloralCityFL	EconfinaFL	0.277	0.27198
FloralCityFL	InvernessFL	-0.02921	-0.0274
FloralCityFL	PalatkaFL	0.75522	0.74631
FloralCityFL	DocThomasFL	0.72936	0.72045
Chipola_River	HoustonTX	0.54639	0.54545
Chipola_River	RedRoadFL	0.83576	0.83129
Chipola_River	ParklandFL	0.82235	0.81711
Chipola_River	GainesvilleFL	0.96387	0.96226
Chipola_River	EconfinaFL	0.30842	0.30729
Chipola_River	InvernessFL	0.92318	0.92
Chipola_River	PalatkaFL	0.40387	0.40206
Chipola_River	DocThomasFL	0.81428	0.80952
HoustonTX	RedRoadFL	0.82702	0.82224
HoustonTX	ParklandFL	0.81382	0.80826
HoustonTX	GainesvilleFL	0.95891	0.95696
HoustonTX	EconfinaFL	0.37279	0.37037
HoustonTX	InvernessFL	0.92083	0.91729
HoustonTX	PalatkaFL	0.49017	0.4874
HoustonTX	DocThomasFL	0.80507	0.8
RedRoadFL	ParklandFL	0.18057	0.17885
RedRoadFL	GainesvilleFL	0.84098	0.83613
RedRoadFL	EconfinaFL	0.492	0.48938
RedRoadFL	InvernessFL	0.79906	0.79263
RedRoadFL	PalatkaFL	0.69934	0.69159
RedRoadFL	DocThomasFL	-0.02712	-0.02798
ParklandFL	GainesvilleFL	0.83733	0.83158
ParklandFL	EconfinaFL	0.5002	0.49587
ParklandFL	InvernessFL	0.79606	0.78873
ParklandFL	PalatkaFL	0.69834	0.68966
ParklandFL	DocThomasFL	0.04539	0.04514
GainesvilleFL	EconfinaFL	0.31398	0.31472
GainesvilleFL	InvernessFL	0.17252	0.17241
GainesvilleFL	PalatkaFL	0.84025	0.83516
GainesvilleFL	DocThomasFL	0.82874	0.82346
EconfinaFL	InvernessFL	0.29185	0.29108
EconfinaFL	PalatkaFL	0.23407	0.23176
EconfinaFL	DocThomasFL	0.46485	0.46227
InvernessFL	PalatkaFL	0.80016	0.79348
InvernessFL	DocThomasFL	0.78627	0.77941
PalatkaFL	DocThomasFL	0.68417	0.67604
